# Design and Implementation of Multiple Music System Based on Internet of Things

**DOI:** 10.1155/2022/3908188

**Published:** 2022-05-30

**Authors:** Mengchen Xu

**Affiliations:** Xi'an Conservatory of Music, Music Education Department, Xi'an 710061, Shaanxi, China

## Abstract

With the rapid development of social economy and Internet of Things, the society has entered the era of networking, digitalization, and intelligence, bringing great convenience to people's life; Internet of Things music system also has begun to get people's extensive attention. Due to the influence of such factors as strong music professionalism, complex music theory knowledge, and diverse changes, it is difficult to identify music features. In order to strengthen the user's personal experience of the music system, the multimusic systems are interconnected through information technology to realize the connection between objects and people. The system uses an embedded processor to realize the central control module and then according to network standard the sensor network is built, through radio frequency identification (RFID) technology for light, sound, infrared sensor, temperature, and other sensors for information reading. Music selection logic is designed based on the theory of music psychology and user behavior log, so as to select the best music for users to improve their mood and improve their life quality and work and study efficiency. At the same time, the system uses voice recognition technology to enhance user interaction, through the system, to provide the website to share their own music data and comments on songs and view song information, and the system runs stably and can collect high quality music signals and correctly identify the characteristics of music form and emotional characteristics.

## 1. Introduction

With the rapid development of computer application and Internet technology, Internet of Things has been formed based on the expansion and extension of the Internet [1–4]. From the information transmission between computers at the beginning to the interconnection between objects, the Internet has jumped out of the traditional communication between computers and turned to the information interaction between all things. At present, all technologies and information interaction related to computers and the Internet can be classified as the Internet of things, and the Internet of things was born. Internet of Things technology can be seen everywhere in our life, such as the Internet of vehicles, smart home, smart city, and so on, all of which are based on the ideas and technologies of the Internet of Things [5–8]. They all have something in common, which are at the heart of the Internet of Things: networks and sensors; network is the bridge connecting each object, the sensor is the tentacle sensing each object, and the sensor collects the attribute information of each object, and this information is transmitted to the computer through the network, and the computer manages and analyzes this information to achieve the desired results.

The concept of the Internet of Things and related technologies provides the physical foundation and guarantee for the Internet of everything, and various industries or fields are also exploring how to further develop in the tide of informatization with the help of the power of the Internet. With music as a widespread form of human society, research related to music and Internet of Things is in the ascendant. Music has been accompanied by the development of individuals, society, and education. It not only effectively records and inherits the brilliant civilization of human beings, but also cultivates and promotes the perceptual thinking ability of human beings in a unique form of sound expression. With the rapid development of the Internet, the improvement of music production technology, the acceleration of creation cycle, and the maturity of compression technology, people can easily release their music works on the Internet, making more and more music works appear on the Internet. As an important medium for obtaining resources, the Internet can provide users with diversified services, making people have more and more channels for acquiring music and more and more music information. However, this has led to the problem of “information overload,” where users often have to spend more time and energy trying to find the music they like. At present, various music teaching software are competing to appear in the public's vision, but there are very few system software that really meet the actual music classroom teaching application needs. There are still some problems in music classroom teaching, such as uneven development of digital music classroom teaching, relative lack of teaching resources, single teaching driving, and lack of interaction between teachers and students; teaching content is not rich enough; it is of great practical significance to design and implement a teaching system that meets the actual teaching needs of music class.

Based on wireless transmission, multichannel audio processing, digital power amplifier, switching power supply, information security technology research and development, with intelligent control, cloud music playback, more mobile terminals support multimusic systems, remote control, and use Internet of things technology to realize the perception, transmission and identification of music signals. At present, the system has been successfully launched after design, implementation, testing, and cloud server deployment. The current platform runs stably. It not only provides users with music resources, but also brings them good use experience.

Based on the current situation of music classroom teaching in the background of digitalization and information age, this paper analyzes many problems existing in the current music classroom teaching process and completes the design and implementation of multiple music system based on Internet of Things according to the characteristics of music classroom teaching. At first, the concept of Internet of Things technology and diverse music is described. Then, the system multikey technology and the multimusic system design scheme are set up. Finally, the multimusic system is designed as a whole, and the intelligent, digital, and visual management of music teaching is realized. The motivation of this paper is that a music feature recognition system based on Internet of Things technology is designed to realize the perception, transmission, and recognition of music signals by using Internet of Things technology, so as to meet the personalized needs of users.

## 2. Analysis and Overview of Key Technologies

The traditional music development model has gradually developed from offline to online, and the current music platform is music as the core [9, 10]. At the same time, under the huge market demand, many music platforms spring up like mushrooms, to meet the personalized needs of different users for music. Internet of Things planning is a management model that relies on modern information technologies such as the Internet, cloud computing, and big data to realize capital accommodation and provide new businesses such as payment and information intermediary. It is the product of the integration of Internet technology and communication management industry. Its main core is the Internet. The Internet of Things is the expansion and extension of the Internet, which extends to objects through two-dimensional code, scanning technology, sensing technology, and other means, realizing the integration and sharing of information resources. Nowadays, Internet of Things technology can be seen everywhere, such as RFID technology, sensor technology, wireless transmission technology, and so on, which are typical representatives. The applications range from the packaging bags of food in daily life to the Space Shuttle, and its scenarios are widely used in medical care, logistics, public safety, environmental protection, and other fields. Internet of Things technology includes the following technologies:

1. Sensor technology: Sensors are usually used as sensing nodes of the Internet of Things to sense and collect information of objects. It has the characteristics of reliability and anti-interference and is an important part of the Internet of Things.
2. Network structure: The Internet of things network structure is composed of four layers: sensor network layer, transmission network layer, middleware layer, and application and service layer. Each part is responsible for different functions and ultimately ensures that the data collected by the sensor node can be transmitted to the upper computer.
3. Recognition technology: Identification technology is commonly used and relatively important in the Internet of Things, including RFID tags and two-dimensional codes, which are often used in logistics and goods. Recognition technology changes the tedious process of traditional object recognition. It can identify objects through a simple two-dimensional code or RFID tag, which greatly improves the efficiency of items and commodities retrieval and classification as well as the speed of location search.
4. Data processing technology: With the increase of nodes in the Internet of Things, the amount of information is bound to explode, so how to deal with so much data is also a problem derived from the Internet of Things technology. As the amount of data increases, “cloud computing” will be a powerful backing for the Internet of Things to process massive data [11–14].

The purpose of preweighting is to flatten the spectrum by elevating the high frequency part of the signal, so that the spectrum and channel parameters of the signal can be analyzed with the same standard throughout the frequency band.

(1) $$\begin{array}{c} H\left(z\right)=1-\mu z^{-1}, \end{array}$$

where *μ* is the preweighting coefficient; the typical value in speech signal processing is 0.94.

In speech signal processing, rectangular window and Hamming window are generally selected for framing, and the window function of rectangular window is

(2) $$\begin{array}{c} h\left(n\right)=\left\{\begin{array}{c} 1,\;0\leq n\leq N-1,\\ 0,\;n=\text{else}. \end{array}\right. \end{array}$$

The fast Fourier transform (FFT) of the *n*-th musical input signal is used to calculate the spectral energy *E*_*n*_ of the signal.

(3) $$\begin{array}{c} X_{n}\left(e^{j\omega }\right)=\sum_{m=0}^{N-1}x_{n}\left(m\right)e^{-j\omega m},\\ E_{n}=\sum_{k=1}^{N}{\left|X_{k}\right|}^{2}\;\left(1\leq n\leq L\right), \end{array}$$

where |*X*_*k*_| is the modulus of the spectrum, *N* is the number of points of FFT transformation, and *L* is the total number of frames.

After calculating the spectral energy, the next step is to select a threshold for endpoint detection. After optimization, the spectrum energy *EY*_*i*_ of the current frame is

(4) $$\begin{array}{c} EY_{i}=\left(1-\theta \right)E_{r}+\theta E_{r+1}, \end{array}$$

where *θ* is the weight factor, *r*=*α* · *L*,  0 < *α* < 1. If the *EY*_*i*_ is greater than the threshold *EY*_*i*_, the *i*-th frame is a speech frame; otherwise, it is a nonspeech frame, and the threshold *Yu* is

(5) $$\begin{array}{c} Yu=\beta \cdot H_{\text{mid}}. \end{array}$$

The most classical algorithm for detecting onset points first estimates the expected value of complex spectrum at the same time and then calculates the distance between the expected value of complex spectrum and the actual value

(6) $$\begin{array}{c} h\left(n\right)=\left\{\begin{array}{c} D\left(t\right)=\frac{1}{N}\sum_{k=1}^{N}{\left|X_{k}^{\prime }\left(t\right)-X_{k}\left(t\right)\right|}^{2},\\ X_{k}^{\prime }\left(t\right)=\left|X_{k}\left(t\right)\right|e^{j\varphi_{k}^{\prime }\left(t\right)},\\ \varphi_{k}^{\prime }\left(t\right)=\frac{\partial^{2}\varphi_{k}\left(t\right)}{\partial t^{2}}, \end{array}\right. \end{array}$$

where *X*_*k*_′(*t*) is the expected value of the complex spectrum, and *φ*_*k*_′(*t*) is the second-order phase difference function.

This paper optimized the calculated peak points, screened and eliminated some wrong or unreasonable peak points, and defined the screening function as *d*(*t*)

(7) $$\begin{array}{c} d\left(t\right)=\lambda_{0}D_{\text{Median}}+\lambda_{1}D_{\text{Mean}}, \end{array}$$

where *D*_Median_ and *D*_Mean_ are the median and mean of the data sample, respectively, and *λ*_0_ and *λ*_1_ are scale factors, respectively. Augmentation *λ*_1_ can prevent detection of some peak points with very small amplitude. Next, define the detection function as *C*(*t*)

(8) $$\begin{array}{c} C\left(t\right)=D\left(t\right)-d\left(t\right). \end{array}$$

Gaussian model, the most widely used model, is used as a classifier to train two GMM models from singing segment data and accompaniment segment data, respectively, which are recorded as singing segment model and accompaniment segment model. The frame coefficient calculated from a partition is denoted as {*L*_1_, *L*_2_,…, *L*_*N*_}, and then

(9) $$\begin{array}{c} \text{Partition }=\left\{\begin{array}{c} \text{Song section},\;\text{When}\sum_{k=1}^{N}p\left(\frac{L_{k}}{{\text{GMM}}_{1}}\right)\geq \sum_{k=1}^{N}p\left(\frac{L_{k}}{{\text{GMM}}_{2}}\right),\\ \text{Accompaniment section},\;\text{When}\sum_{k=1}^{N}p\left(\frac{L_{k}}{{\text{GMM}}_{1}}\right)<\sum_{k=1}^{N}p\left(\frac{L_{k}}{{\text{GMM}}_{2}}\right). \end{array}\right. \end{array}$$

In addition to the Internet of Things technology, the design and implementation of multiple music system should also include the following two key technologies:

1. Simple speech recognition algorithm. The system only needs to identify from the user name some of the basic vocabulary, such as a song, the next song, and changing the background music pattern. The method consists of four steps: feature extraction, template training, template classification, and decision. The signal generated by other information and the code book, that is, the encoder itself, has the ability to distinguish.
2. In terms of the selection of music features used for retrieval, in the early stage, the results of speech signal processing were mainly followed, and the basic time domain and frequency domain features or MFCC features related to the perception characteristics of human ear and hearing were adopted. These features were the features of signal processing level, and the effect was not good when used for music retrieval. Later studies found that the semantic features of music such as melody, rhythm, and timbre can better represent music. At present, most studies use melody as the music feature, because the melody is the most sensitive part of the human ear for music and does not change due to the speed of the play, playing a musical instrument is one of the most important music similarity judging criteria. After repeated research, this paper selected pitch as the musical feature; the reciprocal of pitch cycle is pitch. In the following chapters of this paper, all the musical features mentioned refer to the pitch features of music.
3. Considering that the huge amount of target music data in the music database brings great difficulties to the construction and retrieval of the music feature database, according to the experimental statistics of this paper, music songs have some customary rules, such as the beginning, middle, and end of a song are pure music accompaniment without vocal singing. and songs are generally divided into two paragraphs. The two sections are similar in melody and rhythm, but the lyrics are slightly changed. According to these rules, the target music in the music library is segmented; that is, the useless parts of the whole song are removed, such as prelude, interlude, repeated song section, and end play, leaving only the first song section sung by the singer. After segmentation, the length of the song segment only accounts for about 1/3 of the whole song, the construction of music feature database, and feature matching.
4. Because the target music in the database is multitone music and contains complex background accompaniment, these background accompaniments bring great interference to the extraction of music features, so that the basic pitch extraction algorithm based on time domain or frequency domain cannot extract music features from the target music. This paper studies the existing pitch extraction algorithm based on the fundamental frequency matrix, and the pitch period extraction algorithm is based on the quasi periodicity of the signal short-time Fourier transform. After the short-time power spectrum of the signal is obtained, the peak value is screened, and the most reasonable pitch frequency value is determined after the fundamental frequency matrix is constructed and calculated. But the magnitude of the peak filter is too large, the algorithm in the fundamental frequency matrix does not take into account and when constructing the fundamental frequency matrix, the situation that the fundamental frequency matrix cannot be constructed when the harmonic number is 0 is not considered, and the fundamental frequency matrix is still constructed and calculated when the harmonic number is high, the periodicity is obvious, and it is easy to extract the fundamental frequency, so useless calculations are carried out. On this basis, this paper improves and perfects the pitch extraction algorithm based on the fundamental frequency matrix so that the algorithm can still extract the pitch features from the target music without constructing the fundamental frequency matrix, and reduces the computational complexity.
5. Music feature matching and retrieval technology: DTW algorithm, which is widely used in the field of speech recognition, is a kind of algorithm that transforms global optimum into local optimum and has the performance of time alignment and obtains good results in speech recognition [15–18]. However, the DTW algorithm has some defects when used in music retrieval: The DTW algorithm starts from the beginning of the song, but the user's hum input is very random, not necessarily at the beginning of the song, causing high computational complexity; tonal error cannot be overcome. On this basis, the DTW algorithm for music retrieval is summarized, and some improvements are made to the traditional DTW algorithm, so that it can overcome the pitch error of humming input, reduce the matching time, and optimize the performance of feature matching. After fully analyzing and studying the content-based multimusic retrieval, the overall function design of the multitone music is realized by programming.

## 3. Multimusic System Module Design

Considering the application scope of Internet of Things music system, the design of this system should follow the principles of professionalism, stability, interaction friendliness, timeliness, and scalability, as detailed below:

1. Professionalism. Specialization is one of the key development principles that should be followed when developing. The application scope of the system is mainly classroom music teaching, which is the application scene with high professional requirements. Music itself attaches great importance to sensory experience and quality. If the system in the professional level cannot reach the due level, first of all, for a teacher with professional quality, when using the system, there will be a big discount, and the system can not play a good role. Secondly, for students who just start to learn professional knowledge, it may affect the learning of professional knowledge due to the lack of professional system and misunderstanding of professional knowledge, thus seriously affecting the quality of teaching. Based on the above considerations the professional system should be maximized, in order to ensure accuracy and improve the quality of the system.
2. Stability. Stability is very important for any software or system, and good stability greatly improves the user experience. Compared with other subjects, music teaching is more random. Music teachers may need to improvise a simple music score at any time and then show it to students in the way they want. At the same time, the rules of music itself are complex and changeable, which requires the music teaching auxiliary system to have high fault tolerance and stability. Therefore, when designing the digital music classroom teaching assistant system, we should fully consider various situations to ensure the stability of the system.
3. Interactive friendliness. The friendly interface interaction can greatly improve the user's interest in use. The main user group of the system is music classroom and students; most of the users do not have good computer operation technology literacy, especially the older teachers, so the design process of the system should always be based on the user, to achieve simple operation and rich functions. In addition to the friendly operation of the system, the interface layout and color collocation should also be improved to enhance the aesthetic feeling of the system.
4. Timeliness. Timeliness is also one of the important design principles of digital music classroom teaching assistance system. Here, timeliness mainly refers to the idea that the results of user operations should be responded within the time that does not affect the effect of system functions. Digital music classroom teaching assistant system involves a large number of interactions; some processing tasks are relatively large; in order to achieve better use effect, we need to do special processing for these operations and shorten the response time, to achieve better sensory effect.
5. Scalability. Digital music classroom teaching assistant system is still in the attempt stage; mainly the functions have a demo piano, music editing, and music; there is a great extension of space on the function; the music teaching is also constantly in the development and evolution, in order to adapt to the function of the late development and change the way of music teaching.

### 3.1. System Architecture

**Figure 1:**
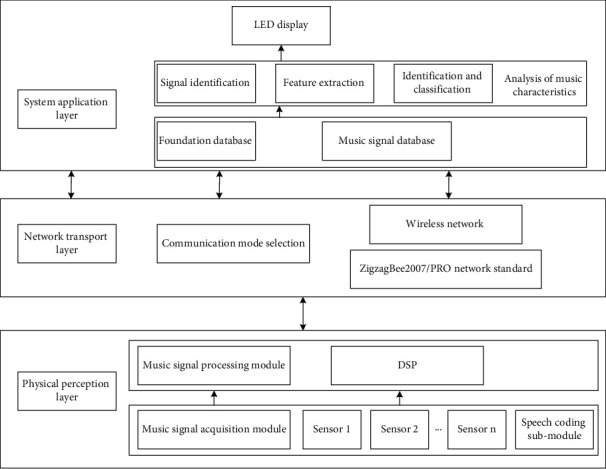
The overall framework of the system.

The central control system design module is to recognize the collected sensor information, including simple speech recognition, infrared information, etc. and through a certain algorithm, select the songs that compound the scene at that time Wireless audio transmission module is used to receive the song information transmitted by the central control system through the Wi-Fi module on the audio, so as to play it out. The design module of user sharing and communication website is used for users to download their favorite songs, share with other users, and leave comments and so on. Music feature recognition system based on Internet of Things technology is mainly composed of physical perception layer, network transmission layer, and system application layer. The overall structure block diagram of the system is shown in Figure 1. The physical perception layer mainly includes music signal acquisition module and music signal processing module. The music signal acquisition module identifies the required music signals through the sound sensor acquisition system in different positions and transmits the collected music signals to the music signal processing module, which uses the DSP processor to process music signals. The network transmission layer transmits the data collected and processed by the physical awareness layer to the system application layer through wireless network communication. The application layer of the system collects music signals to form a music signal database. After the feature extraction and feature recognition classification of music signals in the music feature analysis module, the results of music feature recognition are displayed by LED.

### 3.2. Design of Data Acquisition Module

The music acquisition submodule consists of sound sensors installed in different functional positions, which are responsible for collecting original music signals. A capacitive electret microphone is built into the sound sensor, which is converted by A/*D* converter and transmitted to the voice coding submodule. The speech coding submodule is mainly responsible for the high-fidelity lossless compression of the original music signal, converting the music signal into transmittable data information, and then transmitting it to the music signal processing module.

The photo sensor contains a high-precision photocell. When a constant pressure is applied in the opposite direction to both ends of the cell, any light striking it causes it to release electrons. As a result, the higher the intensity of the light, the greater the current in the cell. Simply put, the photosensitive sensor is to use the photosensitive resistance affected by the light intensity and the principle of resistance changes to send the robot host light intensity analog signal. We have the data collector to transmit light sensor information according to the predefined protocol (for example, in this system the voltage value is divided into five grades, with digital *l* ∼ 5, thus showing a different light intensity value) to the central control system, the latter for processing the information, so as to complete the information acquisition of light feeling.

### 3.3. Design of Central Control System

The software system mainly supports various network protocols data reading from the hardware, data transfer from the application program to the device file and data back to the application program request, and error detection and processing of the device. Photosensitive sensors, temperature sensors, infrared sensors, etc. installed at home are used to detect indoor environmental changes to play corresponding music. Because the signal forms collected by the sensors are different, it is necessary to effectively process the signals, and then transmit them to the CPU in the form of interruption to make corresponding processing, and then send out the control signal.

### 3.4. Design of User Sharing Communication Platform

**Figure 2:**
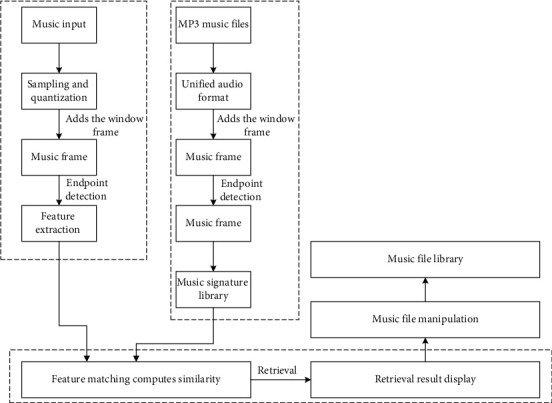
Logic flowchart of multimusic system.

Song share comments module contains system various predefined categories and contains a simple search function; at the same time users can share their own songs and also can comment on the song, so it is convenient for sharing and communication between users. WAMP integrated environment is used on the server side, and the database mainly contains two tables: User and music. Navigation from page to page is accomplished, and then the system array GET is used for extraction. The logic flowchart of multimusic system is shown in Figure 2.

## 4. System Testing and Analysis

**Figure 3:**
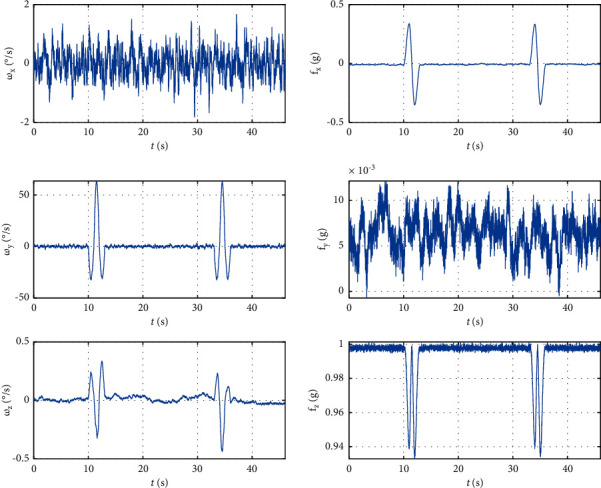
Time domain waveform of music signal.

Figure 3 is the time domain waveform signal, As the target music is strongly interfered by the background accompaniment, it can be seen from the figure that the periodicity of the music signal has been seriously damaged. When the short-time autocorrelation function method, average amplitude difference function method, short-time power spectrum method, and cepstrum method are used for detection, the result is that the frame signal has no pitch. Because of these reasons that cannot be ignored, these basic pitch extraction algorithms based on time domain and frequency domain cannot extract the pitch of singer's singing voice from multitone music with background accompaniment.

**Figure 4:**
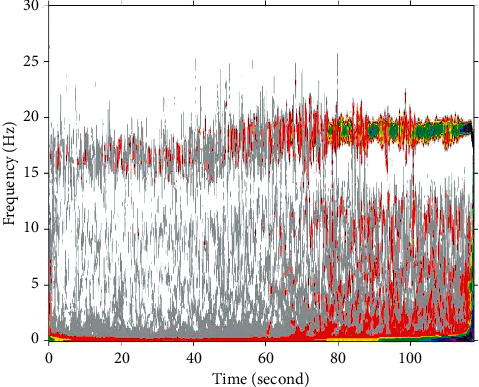
Matching of PWM mixed light and playing music.

When users search the page, they first need to show some popular songs to users and provide them with some popular recommendations, so a recommendation system is very important for this system. Currently, recommendation systems have been widely applied to a lot of fields [19–24]. The method of popular recommendations is mainly determined by the number of times the songs are listened to. When a song is listened to by the user and not marked as a dislike, the popularity of the corresponding song is increased by one. At the same time, according to the user's favorite type of registration, popular recommendations are made for users. The matching of PWM mixed light and playing music is shown in Figure 4.

**Figure 5:**
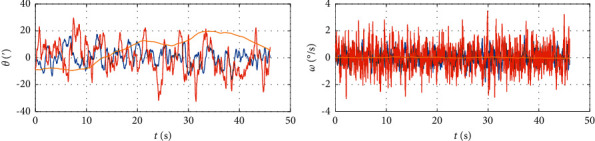
Music signal acquisition results.

By merging the parameters with the dynamic parameters, you can remove the heads and tails. After preprocessing with the algorithm in this paper, we need to be able to effectively represent the characteristics of audio, with good discrimination. Secondly, good independence should be maintained between features. Finally, our coding process should conform to the standard, to ensure the calculation of simple, quick results for analysis. The system sound sensor collected music signals from three different locations in a monitoring area, as shown in the figure. As can be seen from the figure, the music signal curve collected by the system is smooth, without burrs and signal interruption, indicating that the system runs stably and the music signal collected is of good sound quality. The music signal acquisition results are shown in Figure 5.

**Figure 6:**
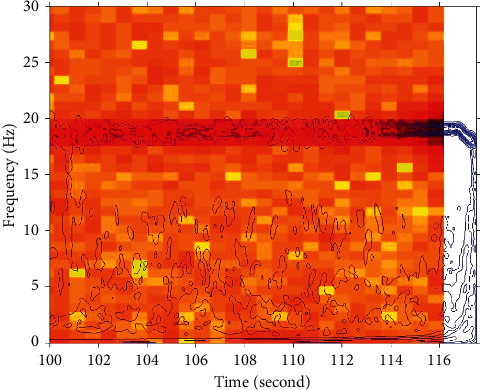
Comparison of basic pitch extraction algorithms.

After testing, the functions of the system have been realized and verified. Through the system function test, from the recommendation on the results, you can see the system can provide users with better recommendation results, based on the real data sets, according to users recommendation songs of training sets; in the test set user accuracy reached 75%, conforming to the level of user acceptance. Therefore, in the future application, the system can be applied to music recommendation to provide users with music recommendation services. The comparison of basic pitch extraction algorithms is shown in Figure 6.

**Figure 7:**
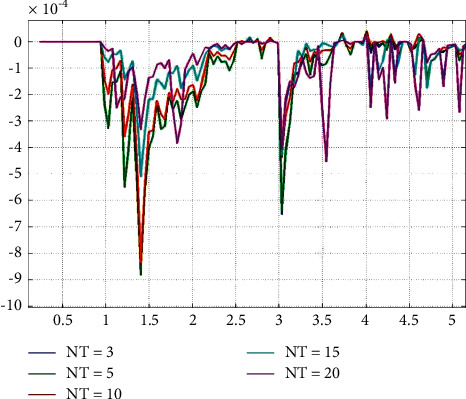
Stability index as a function of frequency.

In order to make the subsequent update and calculation more efficient, the system needs to be arranged on machines with better performance or distributed platform in the future work. In the operation of the system, some relatively large tables need to be accessed frequently, so the method is to maintain a table for each user when logging in to the system and then update the total table once when exiting the system. In this way, only one read and write to the total table is needed, which improves the system performance. Here, for example, the user's behavior table, the user's neighbor table, and the user's recommendation table are all used in this way. The stability index as a function of frequency is shown in Figure 7.

**Figure 8:**
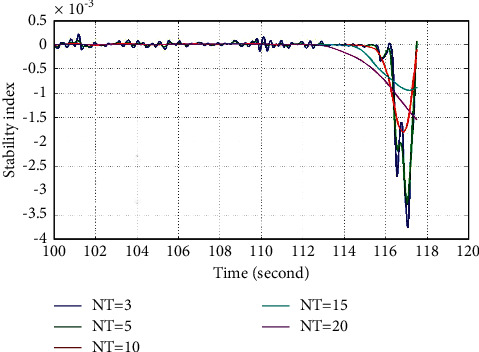
Stability index as a function of time.

Through the test of each module of the system, the final test results verify the concrete realization process of all the functions of the system and ensure the stable operation of each functional module in the system. The core modules include piano playing submodule, music score editing submodule, music score preview and saving submodule, music score display control submodule and music score sound effect processing submodule, which provide technical support for music classroom teaching, simplify teachers' teaching process, and reduce the difficulty of students' actual learning and application. The stability index as a function of time is shown in Figure 8.

**Figure 9:**
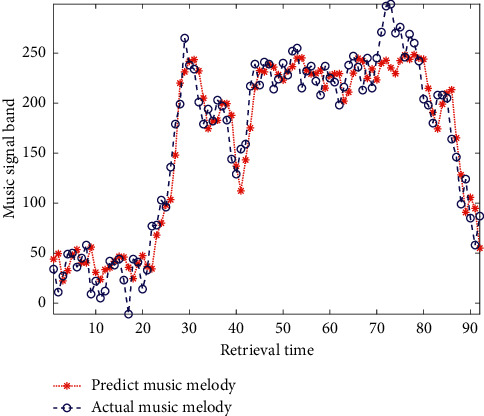
Analysis of experimental results of the system.

Although the design of our idealized process is smooth, whether all data push can affect students' learning behavior and promote their learning improvement ultimately depends on students' attitude towards learning and their interest in pushing content. Through the investigation, we found that the attitude of music college students to study is not greatly influenced by the surrounding classmates, indicating that the students themselves are very personalized and arranged according to their own needs. The analysis of experimental results of algorithm validity is shown in Figure 9.

## 5. Conclusion

Internet of Things is an important form of social development at the present stage, and it is of great value to promote the modernization of society. With combination of requirements analysis and general design, this article, from the digital and information age background music classroom teaching present situation, analyzes the problems existing in the current music classroom teaching process, based on music classroom teaching characteristic, completing the diverse music system based on Internet of Things. Based on the needs of commonly used music teaching software, combined with the characteristics of actual music classroom teaching, the overall structure design of digital music classroom teaching system is completed. Multiple music relying on the Internet of Things is the design of the system; this paper, through the analysis of the concept of Internet of Things technology and diverse music, puts forward the design scheme of multiple music system and design for multiple music system as a whole, realizes the intellectualization, digitalization, and visualization management of music teaching, and improves the service level and quality of music teaching. It is convenient for teachers and students to learn music anytime and anywhere, and users can find the information they need without the limitation of time and place, providing technical support for music classroom teaching.

Based on the research the content of polyphony music retrieval, on the basis of the theory and algorithms, completed the music signal preprocessing, with the improved pitch extraction algorithm based on the fundamental frequency matrix and the DTW algorithm suitable for music retrieval feature extraction and feature matching of music; music retrieval system was designed and implemented in this paper and completed the experimental evaluation system.

Music teaching belongs to a relatively open teaching subject and has diversity in teaching form. On the whole, the system is still in the basic research and development stage; the developed module is only the most basic part of the whole music teaching; in order to better assist the music classroom teaching, there is still a lot of work to do, including the following aspects:

1. The score editing module needs to be further improved by adding more content to support the processing of more special notes, such as conjunctions, appoggiatura, breathing pauses, grace notes, and performance marks.
2. In the music editing module, more music editing methods will be added to facilitate teachers' real-time music editing, such as music editing by recognizing handwritten notes.
3. The system will add more classroom teaching auxiliary modules to support more classroom teaching content, such as rhythm training.
4. The system will develop versions of different operating systems of the system, such as Android tablet PC and iPad tablet PC, so that the system can be applied to more platforms in the later stage to facilitate the interaction between different platforms.

In short, with the continuous development of computer technology and the continuous innovation of music teaching methods, the information music classroom teaching assistant system will follow its pace to constantly update and improve, to provide better and more help for the music classroom teaching under the background of the Internet of Things.

## Data Availability

The data used to support the findings of this study are available from the author upon request.
